# Dilated Cardiomyopathy and Systolic Heart Failure in a Female Patient With Danon Disease

**DOI:** 10.7759/cureus.30803

**Published:** 2022-10-28

**Authors:** Taylor J Stack-Pyle, Joel Shah, Lindsey Stack, Victoria Arcara

**Affiliations:** 1 Otolaryngology - Head and Neck Surgery, University of North Carolina at Chapel Hill School of Medicine, Chapel Hill, USA; 2 Internal Medicine, Liberty University College of Osteopathic Medicine, Lynchburg, USA; 3 Research, Appalachian State University, Boone, USA; 4 Obstetrics and Gynecology, University of North Carolina at Chapel Hill, Chapel Hill, USA

**Keywords:** lysosomal storage diseases, lamp2 mutation, rare genetic disorder, lysosomal storage disorder, heart failure with reduced ejection fraction, dilated cardiomyopathy, danon disease

## Abstract

Danon disease commonly manifests as isolated hypertrophic cardiomyopathy in female patients. The diagnosis is easily missed as it is rare and its pathophysiology is poorly understood. Without early diagnosis and treatment with heart transplantation, cardiomyopathy may progress to heart failure. We present the case of an adolescent female with Danon disease who presented with heart failure with reduced ejection fraction secondary to dilated cardiomyopathy. Her original presentation included a six-month history of shortness of breath, vomiting, and abdominal pain. Further workup revealed pelvic ascites and dilated cardiomyopathy with signs of heart failure. Genetic testing confirmed the diagnosis, revealing a previously undocumented amino acid substitution in the lysosomal-associated membrane protein-2 (LAMP2) gene. The patient received a heart transplant which led to an improvement in her symptoms. The patient’s unique symptomatology illustrates the importance of early cardiac monitoring and transplantation if Danon disease is suspected. This uncommon presentation of dilated cardiomyopathy followed by psychiatric impairment, developmental disability, and unique LAMP2 genetic substitution provides a rare phenotype of Danon disease.

## Introduction

Danon disease is a rare multisystem X-linked dominant disorder associated with a defect in the lysosomal-associated membrane protein-2 (LAMP2) gene [1]. The clinical triad of Danon disease includes skeletal myopathy, intellectual disability, and hypertrophic cardiomyopathy with male patients presenting earlier and with a more severe phenotype than female patients [2]. Female patients typically present with isolated hypertrophic cardiomyopathy which accounts for the majority of known female phenotypes [1,3,4]. As Danon disease is quite rare in the general population, its prevalence and exact pathophysiology are relatively unknown [1,3].

The LAMP2 gene implicated in Danon disease codes for three protein isoforms including LAMP-2A, LAMP-2B, and LAMP-2C which differ in their terminal carboxy lysosomal transmembrane domains [1]. The LAMP2 protein is located abundantly within lysosomal structures and, alongside numerous other isoform-specific functions, has been theorized to help maintain the structural integrity of the lysosomal membrane [1]. It is believed that the loss of LAMP-2B, the primary isoform expressed in muscle, leads to the clinical features of Danon disease. The LAMP2 gene is vital for mediating the process of cellular autophagy and serves as a mediator for autophagosome fusion with lysosomes. Defects in LAMP2 result in an intracellular accumulation of glycogen and vacuoles with autophagic substances, particularly in myocardial and skeletal muscle resulting in cardiomyopathy and skeletal myopathy [1]. LAMP-2B isoform expression in the brain is notably higher in individuals with Danon disease which may account for associated psychiatric disturbances.

Numerous studies have shown a wide array of symptomatology and homeostatic disruption associated with Danon disease. The rapid progression of multisystem dysfunction in patients leads to mortality by the age of 50 in females [3]. Conduction abnormalities, particularly Wolff-Parkinson-White syndrome, are more common in males than females [5]. Ventricular arrhythmias have been reported at a higher frequency in female patients [6]. 

A systematic cognitive and psychiatric assessment of males and females with Danon disease revealed that 69.2% of participants met the criteria for at least one psychiatric illness and 53.8% had two or more psychiatric disorders [7]. Individuals with Danon disease may also display ocular pathology, particularly retinal disease [1]. 

Danon disease is difficult to distinguish from other causes of dilated or hypertrophic cardiomyopathy in female patients. Since female patients regularly progress into advanced heart failure (AHF) by ages 20-40 years, early diagnosis is critical to improve patient survival and facilitate genetic counseling. One study demonstrated the median age at transplant for a cohort with Danon disease was 20.2 years (15.8-27.9 years), with no difference in age between sexes [8]. Median pretransplant left-ventricular ejection fraction for the entire cohort was 30% (range 11%-84%). Heart transplantation outcomes are acceptable in Danon disease with high probabilities of five-year graft survival for males and females suggesting that cardiac transplantation is an effective treatment option [8]. 

We present the case of an adolescent female with chronic and nonspecific symptoms later identified as sequelae of Danon disease with heart failure and a p.Leu272Arg substitution in the LAMP2gene which, to our knowledge, has not previously been documented. 

## Case presentation

A 14-year-old adopted female with a history of anxiety, attention-deficit/hyperactivity disorder (ADHD), and gastroesophageal reflux disease (GERD) presents to her gynecologist for six months of diarrhea, vomiting, abdominal pain, shortness of breath, and intermittent fevers. A pediatrician and pediatric gastroenterologist were also following this patient. Previously, she trialed an increased dose of her selective serotonin reuptake inhibitor for anxiety and omeprazole for GERD, which did not alleviate her symptoms. At the time of presentation to the gynecologist, the patient complained of new left lower quadrant pain and increased nausea. The pain was dull and non-radiating. The patient's mother also reported witnessing episodes of rapid breathing while the patient was sleeping. The patient denied vaginal discharge, foul odor, dysuria, hematuria, constipation, bloody diarrhea, chest pain, or recent illness. Vitals were within normal limits. A physical exam revealed mild epigastric and left lower quadrant tenderness.

**Figure 1:**
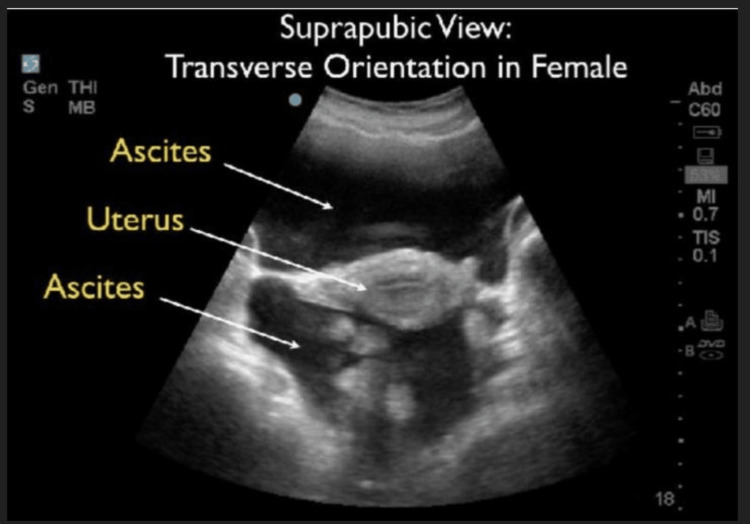
Transverse suprapubic ultrasound view revealing free fluid (ascites) above and below the uterus.

**Figure 2:**
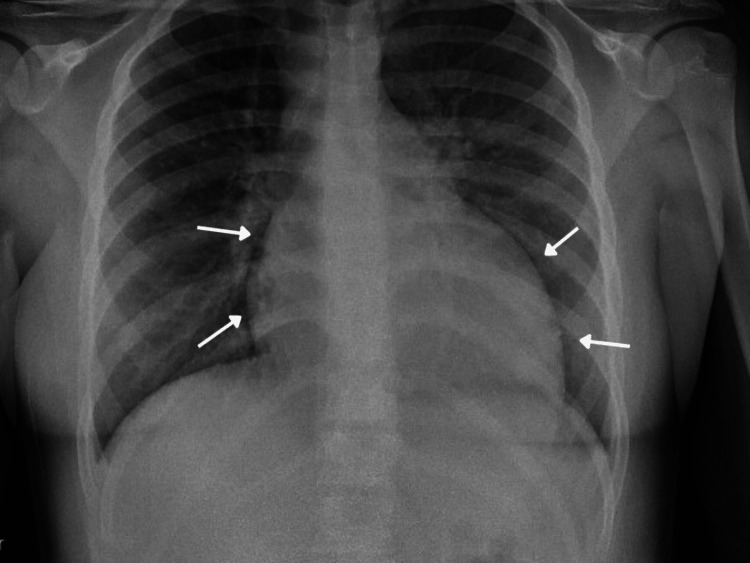
Portable anteroposterior (AP) chest x-ray revealing cardiomegaly. The white arrows illustrate the borders of an enlarged cardiac silhouette.

**Figure 3:**
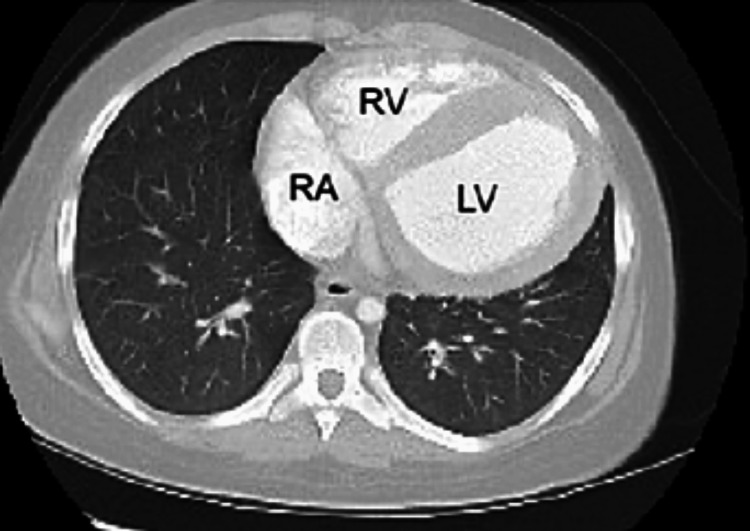
Transverse computed tomography (CT) scan of the thorax illustrating three-chamber dilation consistent with dilated cardiomyopathy. RV=right ventricle, LV=left ventricle, RA=right atrium

A pregnancy test was negative and urinalysis was unremarkable. Ultrasonography revealed a normal uterus and ovaries with free fluid present (Figure 1). The patient was advised to go to the emergency department for the remainder of the workup. At the emergency department, a portable anteroposterior chest x-ray revealed cardiomegaly with a left lower lobe infiltrate (Figure 2). Computed tomography (CT) of the abdomen/pelvis revealed small-volume pelvic ascites and four-chamber cardiomegaly (Figure 3). An electrocardiogram showed sinus rhythm, left axis deviation, right ventricular hypertrophy, and T-wave inversion in lateral leads. The patient's bloodwork demonstrated elevated liver, cardiac, and muscle enzymes, aspartate aminotransferase (AST), 290 U/L, aminotransferase (ALT) 402 U/L; creatine kinase 732 U/L, troponin I 1.83, and B-type natriuretic peptide (BNP) 12,139 pg/mL. A pediatric echocardiogram demonstrated a dilated left ventricle with diminished systolic function, moderate mitral valve insufficiency, hypertrophied right ventricle, mild tricuspid regurgitation, and global hypokinesis. Cardiac measurements were consistent with dilated cardiomyopathy, including interventricular septum thickness diastolic (IVSTd), 1.2 cm; left atrial dimension (LAD), 46.5 mm; left ventricular end-diastolic diameter (LVDd), 6.5 cm; left ventricular ejection fraction (LVEF), 17.5%. Cardiac magnetic resonance imaging (MRI) revealed moderate to severe left ventricular cavity dilatation and severe left ventricular systolic dysfunction with multiple regional wall motion abnormalities. The ejection fraction of the left and right ventricles were 14% and 23%, respectively. There was scarring at the basal right ventricle (RV) septum/anteroseptal to the LV, mid-inferior RV septum, diaphragmatic aspect of the right ventricle, left ventricle, and apical RV free wall, suggestive of myocardial infarctions in the distribution of all three major coronary arteries.

Genetic testing demonstrated a sequence change in the LAMP2 gene, c.815T>G in exon 6, which results in an amino acid change p.Leu272Arg. This sequence change has not been previously documented in patients with LAMP2 gene disorders. Evidence of clinical significance for the p.Leu272Arg substitution is lacking, however, other pathogenic variants in the LAMP2 are associated with X-linked dominant Danon disease.

The patient was transferred to a tertiary care center upon cardiac MRI review and genetic testing. The patient was listed as status 1A for heart transplantation and underwent biventricular assist device implantation. One week later, she underwent orthotopic heart transplantation. The patient was referred to neuropsychiatry by the cardiologist during her admission to the hospital to assess her neurocognitive, behavorial, and emotional function within the context of her diagnosis.

The patient’s initial postoperative course included severe RV dysfunction treated with multiple vasoactive substances, ongoing volume resuscitation, and initiation of inhaled nitric oxide (iNO) for concern of elevated pulmonary artery pressures (PAPs). The post-transplant course included severe delirium requiring Thorazine and benzodiazepines, persistent hyperglycemia treated with insulin infusion, and mild hypertension controlled with an enalapril titration.

Follow-up with neuropsychiatry over the next three years revealed specific learning disabilities with impairment in reading and mathematics. The patient’s feelings of worthlessness, hopelessness, and failure alongside her depressed mood and previous suicidal ideations met the diagnostic criteria for recurrent major depressive disorder. She was also noted to have manipulative and attention-seeking behaviors and somatic symptoms. Psychiatry noted that the patient continually improved upon her learning disabilities and that her conditions were likely related to her diagnosis of Danon disease.

## Discussion

This case presents the rare consideration of dilated cardiomyopathy due to Danon disease as the cause of persistent nonspecific symptoms of systolic heart failure in a pediatric female patient. This presentation is unique given the patient's LAMP2 gene sequence change, c.815T>G in exon 6, resulting in a p.Leu272Arg substitution. Additionally, over 70% of female Danon disease patients present with hypertrophic cardiomyopathy which this patient did not have [4]. Female Danon disease patients regularly progress into advanced heart failure (AHF) by ages 20-40 years, making early diagnosis and heart transplantation critical to patient survival [5]. Current cardiac recommendations for individuals with Danon disease include annual echocardiography with cardiac MRI every one to two years [6,9].

The differential diagnosis for abdominal pain, dyspnea, and ascites in the setting of dilated cardiomyopathy includes myocarditis, rheumatic heart disease, inborn errors of metabolism, and malformation syndromes. This patient did not have evidence of cardiac malformation based on CT and MRI. She did not have a history of strep pharyngitis or other symptoms of rheumatic fever such as arthralgias, nodules, Sydenham's chorea, or erythema marginatum. The patient was transferred to a tertiary care center for further workup where she had a negative cardiac biopsy and viral polymerase chain reaction, ruling out myocarditis.

One study demonstrated the median age at transplant for a cohort with Danon disease was 20.2 years (15.8-27.9 years), with no difference in age between sexes [1,5]. Median pretransplant left-ventricular ejection fraction for the entire cohort was 30% (range 11%-84%). Heart transplantation outcomes are acceptable in Danon disease with high probabilities of five-year graft survival for males and females suggesting that cardiac transplantation is an effective treatment option [1,5].

This case reveals neuropsychiatric manifestations of Danon disease in a female patient which is thought to be uncommon. A systematic cognitive and psychiatric assessment of males and females with Danon disease revealed that 69.2% of participants met the criteria for at least one psychiatric illness and 53.8% had two or more psychiatric disorders [6].

## Conclusions

In conclusion, Danon disease may rarely present as isolated dilated cardiomyopathy with reduced ejection fraction rather than hypertrophic cardiomyopathy in female patients. Danon disease should be included in the differential in pediatric patients with signs of heart failure or congenital heart disease and nonspecific symptoms of unknown etiology. Early identification and cardiac transplantation may be necessary to prevent early mortality due to heart failure in Danon disease patients. Additionally, female patients with Danon disease may develop psychiatric and developmental abnormalities including major depressive disorder and learning disabilities. Though rare, the variations of Danon disease illustrate a unique and educational clinical presentation.
